# A Book Review on Teacher Educator Experiences and Professional Development: Perspectives From the Caribbean

**DOI:** 10.3389/fpsyg.2021.746223

**Published:** 2021-08-26

**Authors:** Yadi Sun

**Affiliations:** Central China Normal University, Wuhan, China

**Keywords:** develop professional teacher educators, teacher educator experience, teacher professional development, teachers' needs for professional development, English language teacher development

Research corroborates that “two qualities which may show teachers the right way in this career journey are their inclination toward education-based research practice and attending to their Continuing Professional Development (CPD) needs” (Derakhshan et al., 2020, p. 1). However, the pendulum has swung much toward teacher professional development (PD) needs, with little attention dedicated to identifying teacher educator professional development needs. Consequently, Jennifer Yamin-Ali's monograph, entitled *Teacher Educator Experiences and Professional Development: Perspectives from the Caribbean*, is a thoughtful voice on teacher educators' professional growth. Reliable and valid research findings constitute an authentic and underappreciated image of a teacher educator from different viewpoints. The specific locus of the research described in this volume is a School of Education, with 34 teacher educators, located in the Caribbean. The book unpacks how such factors as teacher-student interpersonal factors, teacher support, teacher feedback, teacher knowledge and competencies, positive collegial atmosphere, valid course evaluation designs, action research, public advocacy, peer evaluation, self-reflection, among many other factors can contribute to the teacher educators' PD repertoire.

This monograph, a collection of novel and exploratory research articles concentrating on five different aspects of teacher educator PD needs, encompasses five chapters. Triangulating data through questionnaires, interviews, and institutional student assessment of courses from 460 student-teachers from two programs, Chapter 1 explores how student-teachers' voices and feedback can determine the PD needs for teacher educators. The findings document that time management and planning, course evaluation, action research, teaching competencies, specific teaching skills and strategies, self-studies, and personal factors constitute the professional development needs of teachers that should be seriously taken into account. The chapter highlights the role of interpersonal communication factors and emotions to boost personal interaction, communication, and engagement. Yamin-Ali recapitulates that “teacher educators should be instrumental in the development of the instruments used for their formal evaluation, which can consequently provide more reliable and valid results on which to base decision-making as far as their developmental needs are concerned” (p. 33).

In another evidence-based study, Chapter 2 scrutinizes to what extent teacher educators' development is fostered through non-teaching and non-research activities. Collecting data from 19 teacher educators through a closed-answer questionnaire, Yamin-Ali brings to the fore the intricacies of teacher educator roles in the higher education contexts. The author highlights that such needs as “peer evaluation, leading teams, chairing a meeting, coordinating a programme, initiating a community of practice, public advocacy, processes of the department, staffing procedures and resolving conflict” as well as cordial collegial relationships (p. 39) should be taken into consideration to develop professional teacher educators.

Looking at the professional needs of teacher educators from teacher educators' lenses is the focus of Chapter 3. This chapter cogently argues that the practitioners' self-perceived voices play a vital and precious source in identifying their developmental needs. The findings of analyzing the qualitative data gleaned from 14 teacher educators reveal that “knowledge of institutional procedures and collegiality” (p. 71), facilitating personal reflection, improving the opportunities to learn through doing, fostering teamwork, as well as strengthening interpersonal communication with student-teachers and staff.

Like Chapters 2 and 3, Chapter 4 probes deeply into analyzing the detailed narratives and trajectories of four teacher educators' reflections and accounts of professional identity, coping strategies, desires, hardships, and successes to become a teacher educator. The chapter concludes that “self-doubt, a feeling of inadequacy, lack of confidence and a lack of direction surfaced as part of the experience of becoming” (p. 103) a teacher educator. Besides, boosting positive teacher-student collegial relationships and sustained self-reflection can help teacher educators to develop professionally.

Chapter 5 seems to be truly engaging in that the author uses a warm-in-person tone to delineate her personal trajectories to become a teacher educator. The author, as the participant of this empirical chapter, provides instances of how culture and context can pave the way for the PD through encounters with others. The author concludes that “The narrative of my journey toward ‘teacher educator’ has been not just sprinkled with, but punctuated by, serendipitous notes that formed harmonious chords toward a career crescendo” (p. 147).

As a teacher and a teacher educator, I assume that this monograph is enriched not only by the evidence-based research but also by the personalized trajectories of becoming a teacher educator. Although the empirical research studies are contextualized in the Caribbean context, the appraisal of review of the literature and the pertinent theoretical underpinnings about the work and life of teacher educators transcend the local context and deal with the international landscape of becoming and being a teacher educator. Furthermore, the book provides plenty of food for thought because each chapter offers a critical discussion of teacher educators' PD needs through the prism of teacher educators, student-teachers, and the author herself by collecting rich data through open- and close-ended questionnaires, semi-structured interviews, and narratives. Nonetheless, I suggest that, in the new edition, the author can add a detailed description of how she codified, analyzed, and interpreted the qualitative data, bearing in mind such concerns as confirmability, credibility, dependability, transferability, member checking, audit trail, and inter-coder agreement so that the trustworthiness of the evidence-based studies will be enhanced.

As such, this thought-provoking book sheds light on teachers' needs for professional development, contributing to the research field of teacher development as well as to building teachers' academic community. It is useful for student-teachers, teacher educators, academic developers, practitioners and researchers in teacher education, as well as those accountable for administering schools of education and teacher training courses.

## Author Contributions

The author confirms being the sole contributor of this work and has approved it for publication.

## Conflict of Interest

The author declares that the research was conducted in the absence of any commercial or financial relationships that could be construed as a potential conflict of interest.

## Publisher's Note

All claims expressed in this article are solely those of the authors and do not necessarily represent those of their affiliated organizations, or those of the publisher, the editors and the reviewers. Any product that may be evaluated in this article, or claim that may be made by its manufacturer, is not guaranteed or endorsed by the publisher.
